# A phase II trial of S-1 monotherapy in metastatic colorectal cancer after failure of irinotecan- and oxaliplatin-containing regimens

**DOI:** 10.1038/sj.bjc.6603468

**Published:** 2006-11-14

**Authors:** H-C Jeung, S Y Rha, B C Cho, N C Yoo, J K Roh, W J Roh, H C Chung, J B Ahn

**Affiliations:** 1 Cancer Metastasis Research Center, Yonsei University College of Medicine, Seoul, Korea; 2 Yonsei Cancer Center, Yonsei University College of Medicine, Seoul, Korea; 3 BK21 Project for Medical Science, Yonsei University College of Medicine, Seoul, Korea; 4 Department of Internal Medicine, Yonsei University College of Medicine, Seoul, Korea

**Keywords:** colorectal adenocarcinoma, S-1, oxaliplatin-resistant, irinotecan-resistant

## Abstract

This is the first phase II study of S-1 monotherapy for patients with metastatic colorectal cancer after failure of both irinotecan- and oxaliplatin-containing regimens. The initial dose of S-1 was 35 mg m^−2^, administered twice daily for 14 days every 3 weeks. Treatment was repeated until the occurrence of disease progression. Twenty-eight patients were enrolled. S-1 was administered to 21 patients as third-line therapy and to the remaining seven patients as fourth-line therapy. Of 26 evaluable patients, the overall response rate was 14.3% (95% CI, 0.4–28.1), and the disease control rate was 42.9% (95% CI, 23.3–62.4). With a median follow-up period of 227 days, median time to progression and overall survival duration were 91 and 414 days, respectively. The 1-year survival rate of all patients was 60.7%. There was no grade 4 toxicity. Grade 3 haematological toxicities were documented only in two patients. In conclusion, S-1 shows potential as a salvage regimen in heavily pretreated colorectal cancer patients. The twice-daily dose of 35 mg m^−2^ was well tolerated and can be used in designing further combination chemotherapy.

Colorectal cancer is the second leading cause of cancer deaths in Western countries. In Korea, colorectal cancer is the fourth most common malignancy, and its incidence is rising rapidly along with the westernisation of life style (Kim *et al*, 2002). Thirty percent of patients present with advanced disease, and one-half of patients who have undergone surgery eventually develop metastasis. The prognosis of metastatic disease is poor, although palliative chemotherapy has been shown to prolong survival over best supportive care (Glimelius *et al*, 1994). 5-Fluorouracil (5-FU) has been widely used in the treatment of metastatic colorectal cancer, and its modulation by leucovorin increased the response rate to 23%.

With the introduction of irinotecan and oxaliplatin during the last decade, treatment choice for metastatic colorectal cancer has been extended. Irinotecan or oxaliplatin combined with 5-FU resulted in improved tumour response with a moderate survival prolongation (De Gramont *et al*, 2000; Douillard *et al*, 2000; Saltz *et al*, 2000). Moreover, there have been recent trials introducing these agents in adjuvant therapy (Alberts *et al*, 2005; Tyagi *et al*, 2005). This rapid change in treatment strategy will ultimately lead to a considerable increase in the number of patients with recurrent or progressive disease who have been previously exposed to irinotecan and oxaliplatin, and it raises the necessity for salvage therapy for this patient group. Currently, chemotherapeutic option for these patients is quite limited. Cetuximab is regarded to be a promising option in this setting, and capecitabine is under study of alternative dosing method for use in combination regimens, and investigational agents such as bevacizumab have been tried restrictively for selected patients (Cunningham *et al*, 2004; Saltz *et al*, 2004; Gubanski *et al*, 2005; Matin *et al*, 2005).

Oral chemotherapy has advantages in the aspects of pharmacoeconomics and patient preference. Oral fluoropyrimidines are considered to be an alternative to conventional protracted 5-FU infusion as far as they provide comparable efficacy and compliance. A novel oral fluoropyrimidine, S-1, has been developed to improve the therapeutic index of tegafur (FT), and 5-FU level was maintained high in plasma and tumour with less gastrointestinal toxicity by combination with two biomodulators, 5-chloro-2,4-dihydroxypyridine (CDHP) and potassium oxonate (Oxo). S-1 has shown promising activity in untreated colorectal cancer through several phase II trials. And in a phase I study, S-1 led to objective response in heavily pretreated colorectal cancer patients (Chu *et al*, 2004). Based on these results, we conducted a phase II study to evaluate the possibility of S-1 as a salvage option for heavily pretreated colorectal cancer patients who had previously received irinotecan and oxaliplatin.

## PATIENTS AND METHODS

### Patient eligibility

The eligibility criteria were as follows: histologically proven colorectal adenocarcinoma with metastatic, inoperable disease, Eastern Cooperative Oncology Group (ECOG) performance scale 0–2, documented disease progression during or within 6 months after treatment with irinotecan- and oxaliplatin-containing regimens, at least one unidimensionally measurable lesion as assessed by spiral computed tomography outside any previously irradiated area, age ⩾18 years old, life expectancy ⩾3 months, and adequate organ functions (WBC ⩾3000 *μ*l^−1^, platelets ⩾100 000 *μ*l^−1^, haemoglobin ⩾9.0 g dl^−1^, serum creatinine ⩽1.5 × of the upper limit of normal (ULN), bilirubin ⩽1.25 × ULN, and serum aminotransferases ⩽2.5 × ULN). Patients were excluded if they had other active malignancies, brain metastasis, or severe comorbid conditions. After IRB approval, informed consent from all patients was obtained before enrolment.

### Treatment schedule

The starting dose of S-1 was twice daily at 35 mg m^−2^. S-1 was administered within 1 h after meals for 2 weeks, followed by a 1-week rest. The schedule was repeated until the occurrence of disease progression, unacceptable toxicities, or patient‘s refusal. A dose reduction of 10 mg m^−2^ a day was made if ⩾grade 3 haematological or non-haematological toxicity was shown in the previous cycle. Dose re-escalation was not allowed. Patients who required more than 4 weeks of rest for recovery from any toxicity other than alopecia, nausea, vomiting, or anaemia, or who required dose reduction of more than two steps (total 20 mg m^−2^ a day), were withdrawn from the study.

### Evaluation of response and adverse event

Baseline evaluations included a complete medical history, physical and radiologic examinations, performance status, complete blood count (CBC), and biochemistries. During treatment, patients were evaluated with a weekly CBC. Physical examination, performance status, biochemistries were re-evaluated before each subsequent cycle. Imaging studies for lesions were repeated every two cycles.

Treatment response was evaluated according to the guidelines of the Response Evaluation Criteria in Solid Tumors (RECIST) Committee. Patients were considered to be assessable for response if they had evidence of early disease progression clinically or radiologically before two cycles, or if they had received a minimum of two cycles of treatment with at least one tumour measurement. If a patient was documented as having a complete response (CR) or a partial response (PR), a confirmatory evaluation was performed after 4 weeks. Time to progression (TTP) was defined as the interval from the first day of treatment until disease progression, and overall survival (OS) was calculated from the first day of treatment to death. All the patients were evaluated for adverse events weekly. Adverse events were graded according to the NCI-CTC scale (Version 3.0).

### Statistics

Time-dependent variables were estimated with a log-rank test using the Kaplan–Meier method. This study was designed using the Minimax two-stage design (Simon, 1989). Sample size was calculated with 80% power to detect an objective response rate of 15% (P1) and to rule out a response rate of 3% (P0). The first stage was determined with 18 patients, and the criterion for continued accrual was the observation of at least one tumour response. The second stage was planned to accrue a further 10 patients with two more responses. The required number of patients was determined to be 28.

## RESULTS

### Patient characteristics

A total of 28 patients entered the study between July 2004 and October 2005. Baseline patient characteristics are listed in Table 1. Twenty-six patients were evaluable for tumour response. Two patients refused treatment after the first cycle and withdrew the consent. The median age was 56 years. All but four patients had undergone prior resection of their primary tumour. Liver and lung were the most common sites of measurable lesions, and peritoneal seeding was the main site of non-measurable lesions. The average diameter of target lesion was 26 (range 10–99 mm) and the median number of measurable lesions per patient was three (range 1–8).

Table 2 summarises the prior chemotherapy our patients received. S-1 was administered as third-line therapy to 21 patients and the remaining seven patients received S-1 as fourth-line therapy. The median time from documentation of disease progression (or relapse) of previous regimen to S-1 treatment was 21 (range 10–84) days. The median cycle number of previous chemotherapy was 18 (range 7–33) per patient, and that of regimens containing irinotecan or oxaliplatin was 6, with a median dose intensity of 0.98.

### Treatment outcomes

A total of 125 treatment cycles (median 2, range 1–11) were administered. The median daily dose administered was 120 (range 100–150) mg. Two patients were subjected to dose reduction owing to adverse events. Seven patients voluntarily delayed their subsequent cycle by 1 week without any significant toxicity. The median dose intensity of all the patients was 317 (range 205–327) mg m^−2^ week^−1^. Seventeen patients (60.7%) were switched over to the next chemotherapy regimens after disease progression was documented.

### Efficacy

Of the 26 patients evaluable for response, one CR and three PRs were achieved, and eight patients had stable disease. The overall response rate was 14.3% (95% CI, 0.4–28.1) and disease control rate was 42.9% (95% CI, 23.3–62.4) by intent-to-treat analysis (Table 3). All objective responses were documented after two cycles of treatment and lasted 126, 195, 248, and 204+ days, respectively. Metastatic sites in the liver and lung had a response rate of 10.2 and 23.3%, respectively, whereas other sites showed no objective tumour shrinkage. No difference in efficacy was observed with respect to the size or the number of lesions.

### Survival

**Figure 1:**
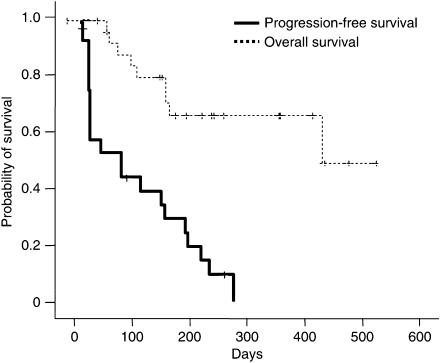
Survival analysis of all patients (*n*=28).

With a median follow-up duration of 227 (range, 28–567) days, 24 patients (86%) showed disease progression and nine patients (32%) expired from disease. Median TTP was 91 (95% CI, 9–173) days (Figure 1). Median OS for all patients was 414 (95% CI, 336–492) days, and the 1-year survival rate was 60.7%.

### Safety

No grade 4 adverse event or treatment-related mortality was observed. The most common haematologic adverse event was anaemia without evidence of bleeding, which occurred in 14 patients (50%), with two patients receiving transfusions. Severe neutropenia (grade ⩾3) was reported in two patients. The most common non-haematologic adverse event was nausea, which affected 64% of patients. Adverse events were well managed and self-limiting. Grade 3 adverse event amenable to dose reduction was recorded in two patients, consisting of neutropenia and anaemia combined with neutropenia, respectively. Adverse events associated with treatment are listed in Table 4.

## DISCUSSION

Regimens including 5-FU with irinotecan, oxaliplatin, and bevacizumab have proven effective in the treatment of colorectal cancer as first-line and second-line treatment. However, there have been few reports of satisfactory salvage therapy in heavily pretreated patients. Novel target agents such as cetuximab have been promising for their potential role as salvage therapy (Cunningham *et al*, 2004). However, oral agents could be a promising alternative when taking the patients' general condition and quality of life into consideration.

Capecitabine has recently been tried as salvage therapy, but the efficacy was not satisfactory. Capecitabine showed no objective response in 5-FU-resistant cancer in a phase II trial (Hoff *et al*, 2004). Combination with MMC resulted in a response rate of 15% in irinotecan-resistant cancer, but its efficacy fell to 5% when the cancer was resistant to both irinotecan and oxaliplatin. Other combinations with trimetrexate or irinotecan did not show any additional benefit either (Chong *et al*, 2005; Gubanski *et al*, 2005; Matin *et al*, 2005).

Integrating S-1 in colorectal cancer has been proposed in several phase II studies. These studies reported a response of 24–40% with a median TTP of 5–6 months when used as a first-line treatment (Ohtsu *et al*, 2000; Van den Brande *et al*, 2003; Shirao *et al*, 2004). Our study is the first one investigating the role of S-1 as a salvage treatment in irinotecan- and oxaliplatin-pretreated colorectal cancer. One of our concerns in designing this phase II trial was the predictive efficacy of S-1. Our study was designed to show a response rate of 15%, which is quite high considering the activity of infusional 5-FU and capecitabine in chemotherapy exposed patients. Nevertheless, this response rate is currently the minimum response expected from any new agent targeting second- or third-line treatment for colorectal cancer (Hoff *et al*, 2004; Gubanski *et al*, 2005). Treatment schedule of S-1 was another point of consideration. Japanese trials traditionally adopted 80 mg m^−2^, but their actual dose is inconsistent with BSA (64–80 mg m^−2^) (Ohtsu *et al*, 2000; Shirao *et al*, 2004). Meanwhile, Western studies are BSA consistent. However, in a sole European phase II trial, a dose of 80 mg m^−2^ had to be reduced owing to significant non-haematological toxicity. We adopted 70 mg m^−2^ and a 3-week schedule for the purpose of procuring adequate dose intensity and compliance even in heavily exposed patients to chemotherapy. With this dosage system, we could attain high-dose intensity of 317 mg m^−2^ week^−1^ (97%).

Preclinical modelling gave several evidence that S-1 would be effective even in pretreated colorectal cancer. S-1 showed higher tumour growth inhibition than UFT did in an orthotopic implantation model of colon cancer, and S-1 promoted antitumour activity in chemoresistant cancer cells *in vitro* (Shirasaka *et al*, 1996). In a phase I study, S-1 resulted in objective antitumour activity in heavily-pretreated colorectal carcinoma, and there was a report that S-1 led to a CR in 5-FU-resistant gastric cancer (Chu *et al*, 1994; Tsukioka *et al*, 2001). All of these findings were the basis of our designing phase II trial, and we observed a response rate of near 15%, which imposes a promise of S-1 for heavily pretreated patients. It is difficult to clarify the mechanisms behind the effectiveness of S-1, which are different from those of other 5-FU modulators. Higher plasma or intratumoural concentrations of 5-FU could be obtained from inhibition of dihydropyrimidine dehydrogenase by CDHP compared to simple 5-FU infusion. The inhibitory effect of CDHP on the production of *α*-fluoro-*β*-alanine might be another explanation, as it was reported to reduce the antitumour effect of 5-FU (Cao *et al*, 2000).

From the perspective of survival, four patients who obtained objective tumour response had a median response duration of 200 days, suggesting that antitumour activity of S-1 was durable even in heavily treated patients once response was induced. The median TTP was 91 days, which is comparable to previously reported third-line therapy. It is surprising that OS was more than 400 days. This result might come from patient selection mechanisms irrespective of therapeutic effects of S-1. But, S-1 treatment showed favourable safety profiles even in heavily pretreated setting, resulting in median dose intensity of 97% and over 60% of patients were switched to yet other salvage regimens including cetuximab, capecitabine, or other novel investigational agents after failure of S-1. This suggests that patients could maintain their performance status during S-1 treatment enough to tolerate next regimen and that S-1 rarely affected the patients' compliance and quality of life. It is noticeable that the group previously exposed to capecitabine had a tendency of shorter TTP compared to the capecitabine-naïve group (38 days *vs* 111 days, *P*=0.33). Although not statistically significant owing to the small number of patients, this result implies that resistance to capecitabine may adversely influence the efficacy of S-1, and it should be an eligibility consideration for future clinical trial design.

In conclusion, this first phase II study of S-1 in irinotecan- and oxaliplatin-pretreated colorectal carcinoma demonstrated high-dose intensity, promising efficacy and survival with favourable safety profile. S-1 shows potential activity as a salvage therapy in heavily pretreated colorectal cancer. Dose of 35 mg m^−2^ twice daily is feasible and can be used in designing further combination chemotherapy.

## Figures and Tables

**Table 1:** Patient characteristics

**Patient characteristics**	**Number of patients (%)**
Number of enrolled patients	28
Number of evaluable patients	26
Median age (years) (range)	56 (32–69)
Male: female	15:13
	
*ECOG performance status*	
0–1	8 (28.6)
2	20 (71.4)
	
*Primary site*	
Colon	18 (64.3)
Rectum	10 (35.7)
	
*Histological differentiation*	
Well moderate	25 (89.3)
Poor	3 (10.7)
	
*Prior resection of primary site*	
Yes	23 (82.1)
No	5 (17.9)
	
*Site of measurable lesions*	
Liver	49 (52.1)
Lung	30 (31.9)
Lymph node	12 (12.8)
Abdominal mass	3 (3.2)
	
*Metastatic site per patient*	
1	10 (35.7)
2	12 (42.9)
⩾3	6 (21.4)

ECOG=Eastern Cooperative Oncology Group.

**Table 2:** Summary of prior chemotherapy

	**Number of patients**					**Dose intensity**	**Cycle**
	**Total**	**CR/PR**	**SD**	**PD**	**Post-op^a^**	**Median (range)**	**Median (range)**
Adjuvant							
5-FU+LV	6	—	—	—	6	1.00	6 (2–10)
							
*First line*							
5-FU+LV	3	—	—	—	3	1.00 (0.94–1.00)	6 (6–12)
Irinotecan-based^b^	11	4	1	1	5	0.98 (0.62–1.00)	6 (3–12)
Oxaliplatin-based^b^	14	4	4	1	5	0.96 (0.84–1.00)	6 (2–9)
							
*Second line*							
Irinotecan-based^c^	15	8	5	2	—	0.93 (0.63–1.00)	6 (2–18)
Oxaliplatin-based^c^	13	3	7	2	1	0.98 (0.82–1.00)	6 (2–12)
							
*Third line*							
Irinotecan-based^d^	2	—	1	—	1	NA^e^ (0.94–1.00)	NA^e^ (6–8)
Oxaliplatin-based^e^	1	—	1	—	—	NA^e^ (0.93)	NA^e^ (4)
5-FU+cisplatin	1	—	—	1	—	NA^e^ (1.00)	NA^e^ (2)
Capecitabine-based^f^	3	—	1	2	—	1.00 (0.92–1.00)	3 (2–3)

5-FU=5-fluorouracil; LV=leucovorin; CR=complete response; PR=partial response; SD=stable disease; PD=progressive disease; Post-op=postoperative; NA=not assessable.

a Represents in people who received chemotherapy after radical dissection of all visible lesions (R0 resection) of whether primary or metastasis. These patients do not have measurable lesion, therefore their primary end point of treatment was recurrence.

b All the patients received first-line treatment which were combined with 5-FU continuous infusion modulated by leucovorin.

c All but five patients received second-line treatment which were combined with 5-FU+leucovorin or capecitabine. The five patients were in irinotecan group and received monotherapy.

d Received iriotecan monotherapy, FORFIRI, and XELOX, respectively.

e Median value was not obtainable owing to small patient number.

f Received monotherapy (two patients) and combined with mitomycin-C (one patient).

**Table 3:** Response to treatment

	**N**	**CR**	**PR**	**SD**	**PD**	**NE**	**Response rate (%)**	**Disease control rate (%)**
Overall	28	1	3	8	14	2	14.3	42.9
(95% CI)							(0.4–28.1)	(23.3–62.4)
								
*Metastatic target sites*								
Liver	49	2	3	17	22	5	10.2	44.9
Lung	30	1	6	13	10	—	23.3	66.7
Others	15	—	—	9	6	—	0.0	60.0

N=number; CR=complete response; PR=partial response; SD=stable disease; PD=progressive disease; NE=not evaluable; CI=confidence interval; 95% CI, 95% CI.

**Table 4:** Adverse events per patient

	**Number of patients (N=28)**				
	**Grade 1**	**Grade 2**	**Grade 3**	**Toxicity of all grades (%)**	**Toxicity of grade 3 (%)**
*Hematologic toxicity*					
Anaemia	7	6	1	50.0	3.6
Leucopenia	7	4	—	39.3	—
Neutropenia	4	3	2	32.1	7.1
Thrombocytopenia	5	—	—	17.9	—
					
*Non-hematologic toxicity*					
Diarrhoea	8	2	—	35.7	—
Nausea	16	2	—	64.3	—
Vomiting	9	1	—	35.7	—
Mucositis	9	2	—	39.3	—
Anorexia	16	1	—	60.7	—
Fatigue	7	1	—	28.6	—
Weight loss	5	3	—	28.6	—
Dyspepsia	7	—	—	25.0	—
Skin rash	3	—	—	10.7	—
Itching sensation	4	—	—	14.3	—
Skin pigmentation	9	2	—	39.3	—
Hand-and-foot syndrome	9	1	—	35.7	—
Abdominal pain	10	1	—	39.3	—
Elevated creatinine	2	—	—	7.1	—
Changes in liver function	13	4	—	60.7	—
